# Study of damage control strategy for non-traumatic diseases: a single-center observational study

**DOI:** 10.1186/s40001-022-00823-8

**Published:** 2022-10-01

**Authors:** Fumiko Nakamura, Rintaro Yui, Atsunori Onoe, Masanobu Kishimoto, Kazuhito Sakuramoto, Takashi Muroya, Kentaro Kajino, Hitoshi Ikegawa, Yasuyuki Kuwagata

**Affiliations:** Department of Emergency and Critical Care Medicine, Kansai Medical University, Shinmachi 2-5-1, Hirakata, Osaka 573-1010 Japan

**Keywords:** Acute abdomen, Damage control strategy, Non-traumatic diseases, Open abdominal management, Septic shock

## Abstract

**Background:**

Damage control strategy (DCS) has been introduced not only for trauma but also for acute abdomen, but its indications and usefulness have not been clarified. We examined clinical characteristics of patients who underwent DCS and compared clinical characteristics and results with and without DCS in patients with septic shock.

**Methods:**

We targeted a series of endogenous abdominal diseases in Kansai Medical University Hospital from April 2013 to March 2019. Clinical characteristics of 26 patients who underwent DCS were examined. Then, clinical characteristics and results were compared between the DCS group (*n* = 26) and non-DCS group (*n* = 31) in 57 patients with septic shock during the same period.

**Results:**

All 26 patients who underwent DCS had septic shock, low mean arterial pressure (MAP) before the start of surgery, and required high-dose norepinephrine administration intraoperatively. Their discharge mortality rate was 12%. Among the patients with septic shock, the DCS group had a higher SOFA score (*P* = 0.008) and MAP was lower preoperatively, but it did not increase even with intraoperative administration of large amounts of fluid replacement and vasoconstrictor. There was no significant difference in 28-day mortality and discharge mortality between the two groups.

**Conclusions:**

DCS may be useful in patients with severe septic shock.

## Background

To improve short-term mortality in patients with severe trauma and the deadly triad of metabolic acidosis, blood coagulation disorders, and hypothermia, the usefulness of a damage control strategy (DCS) that includes control of active bleeding and intra-abdominal contamination, simultaneous physiological resuscitation and subsequent radical surgery has been recognized [1–3]. Since the introduction of DCS for acute abdomen in the early twenty-first century, its indications and usefulness have not been clarified [4–8]. DCS for acute abdomen is a multifaceted strategy. If a patient’s general condition is unstable, priority is given to resuscitation, and only source control is performed to shorten the operation time. Then, after the patient’s general condition has improved, anatomical reconstruction, and abdominal closure are performed [9]. The indication for DCS for acute abdomen in our hospital is determined by the surgeon for those patients in whom hemodynamics are extremely unstable despite appropriate resuscitation during and before surgery. The purpose of this study was to review patients with acute abdomen who underwent DCS at our hospital, clarify the clinical characteristics of these patients, and then compare the clinical course and results in the patients with and without DCS.

## Methods

In this retrospective cohort study, we targeted 443 emergency operations for a series of non-traumatic abdominal diseases performed in Kansai Medical University Hospital, Japan, from April 2013 to March 2019. We excluded patients under 15 years of age and those for whom we were unable to perform a second-look operation due to their poor condition. Among the remaining 438 patients, 57 patients were in septic shock and considered to have unstable hemodynamics. We divided them into 26 patients who underwent DCS (DCS group) and 31 patients who did not undergo DCS (non-DCS group) for comparison.

The following patient characteristics were evaluated: age, sex, disease (upper gastrointestinal perforation, lower gastrointestinal perforation, acute intestinal necrosis, strangulated ileus, and others), history of dialysis, history of diabetes, taking steroids, preoperative Sequential Organ Failure Assessment (SOFA) score, preoperative acute disseminated intravascular coagulation (DIC) score [10], serum lactic acid level immediately at the end of surgery, presence of septic shock, infusion and transfusion volume during surgery, norepinephrine (use of 0.2 μg/kg/min or more) during surgery, mean arterial pressure (MAP) at the start of surgery, MAP at the end of source control, and 28-day mortality rate.

In the group comparison between the DCS group and non-DCS group, we compared six factors of the SOFA score, operation time, amount of bleeding, and discharge mortality rate. In addition, in the DCS group, complications of open abdominal management (OAM) (fascial closure impossible, hemorrhagic complications, intestinal fistula, and number of days until closure) were also examined.

Septic shock was defined by The Third International Consensus Definitions for Sepsis and Septic Shock (Sepsis-3) in 2016 as follows [11]. Organ dysfunction could be identified as an acute change in total SOFA score ≥ 2 points consequent to the infection. Patients with septic shock could be identified with a clinical construct of sepsis with persistent hypotension requiring vasopressors to maintain MAP ≥ 65 mmHg and having a serum lactate level > 2 mmol/L (18 mg/dL) despite adequate volume resuscitation.

Source control was defined in the cases of upper gastrointestinal perforation as being when the perforation was closed and in the cases of lower gastrointestinal perforation, acute intestinal necrosis, and strangulated ileus as being when the lesion was resected. After that, intestinal stumps were left in discontinuity using a LigaSure and a linear stapler.

The DCS group underwent source control and OAM. In OAM, the open abdominal wound was treated with local negative pressure wound therapy at -75 mmHg (Renasys EZ MAX; Smith & Nephew GmbH or ABTERA® Dressing Kit; KCIKK, Tokyo, Japan). Our critical care center managed the intensive care of the patients before and after surgery. During the first 24–48 h, a second-look operation was carried out, and additional intestinal resection was performed if the progression of ischemia or a necrotic intestinal tract was found during the procedure. An anastomosis was performed if it was judged possible, but if not possible, a colostomy was constructed. After completing source control and considering the intra-abdominal pressure, the abdomen was closed.

Categorical data are presented as numbers (%) and were compared by Chi-square or Fisher’s exact test as appropriate. Continuous variables are expressed as the median and interquartile range and were compared using the non-parametric Mann–Whitney test. The threshold for significance was a *P* value < 0.05. Logistic regression analysis was used for multivariate analysis. All statistical analyses were conducted using IBM SPSS version 26.

This study was approved by the ethics committee of our institution (approval no.: 2019157).

## Results

As a result of examination of the 26 patients who underwent DCS, all patients had septic shock. There were many cases of lower gastrointestinal perforation and acute intestinal necrosis. In many cases, MAP was low at the start of surgery and required high-dose norepinephrine administration during surgery. The 28-day mortality rate of these patients was 12% (Table 1).

**Table 1:** Clinical characteristics and outcome of patients undergoing DCS

Characteristics	*n* = 26
Age (years)	76 (47–88)
Male, *n* (%)	14 (53.8)
Disease	
Upper gastrointestinal perforation, *n* (%)	1 (3.8)
Lower digestive tract perforation, *n* (%)	12 (46.2)
Acute intestinal necrosis, *n* (%)	9 (34.6)
Strangulation ileus, *n* (%)	3 (11.5)
Others, *n* (%)	1 (3.8)
Dialysis history, *n* (%)	2 (7.7)
Diabetes history, *n* (%)	4 (15.4)
Taking steroid, *n* (%)	3 (11.5)
Preoperative SOFA score	7.5 (2–16)
Preoperative acute DIC score^a^	3 (1–8)
Serum lactic acid level just before the end of surgery (mmol/L)	5.5 (2.3–15.6)
Septic shock^b^, *n* (%)	26 (100)
Fluid and blood transfusion volume during surgery (mL)	3473 (667–7844)
Use norepinephrine ≥ 0.2 μg/kg/min during surgery, *n* (%)	20 (76.9)
MAP at the start of surgery (mmHg)	68 (33–107)
MAP at the end of source control (mmHg)	65 (51–101)
28-day mortality, *n* (%)	3 (11.5)

Categorical variables are expressed as median (interquartile range)

*DCS* damage control surgery, *SOFA* sepsis-related organ failure assessment, *DIC* disseminated intravascular coagulation, *MAP* mean artery pressure

^a^Diagnostic criteria for DIC established by the Japanese Association for Acute Medicine

^b^When the SOFA score rises by ≥ 2 points, condition requires vasopressor to maintain MAP ≥ 65 mmHg and serum lactate level > 2 mmol/L

There were 57 patients with septic shock, of whom 26 comprised the DCS group and 31 comprised the non-DCS group. As a result of comparative study, the DCS group had a lower number of cases of upper gastrointestinal perforation and a higher number of cases of acute intestinal necrosis than the non-DCS group. The operation time and bleeding volume were significantly shorter and smaller, respectively, there were no complications of OAM, and the number of days until fascial closure was 2 days in the DCS group. The preoperative SOFA score was significantly higher in the DCS group (*P* = 0.008), and the preoperative MAP was lower and so severe in the DCS group that the intraoperative MAP did not increase even after a large volume of fluid and a vasoconstrictor were administered intraoperatively. However, there was no significant difference in the rates of 28-day mortality and discharge mortality between the DCS group and non-DCS group (28-day mortality: *P* = 0.499; discharge mortality: *P* = 0.508) (Table 2).

**Table 2:** Clinical characteristics and outcome in patients with septic shock in the DCS and non-DCS groups

Characteristic	DCS (*n* = 26)	Non-DCS (*n* = 31)	*P* value
Age (years)	76 (47–88)	79 (57–96)	0.272
Male, *n* (%)	14 (53.8)	18 (58.1)	0.749
Disease			
Upper gastrointestinal perforation, *n* (%)	1 (3.8)	7 (22.6)	0.043
Lower digestive tract perforation, *n* (%)	12 (46.2)	17 (54.8)	0.514
Acute intestinal necrosis, *n* (%)	9 (34.6)	3 (9.7)	0.021
Strangulation ileus, *n* (%)	3 (11.5)	1 (3.2)	0.221
Others, *n* (%)	1 (3.8)	3 (9.7)	0.391
Dialysis history, *n* (%)	2 (7.7)	2 (6.5)	0.855
Diabetes history, *n* (%)	4 (15.4)	4 (12.9)	0.788
Taking steroid, *n* (%)	3 (11.5)	1 (3.2)	0.221
Preoperative SOFA score	7.5 (2–16)	4 (2–14)	0.008
Respiration	2 (0–4)	1 (0–4)	0.039
Coagulation	1 (0–3)	0 (0–2)	0.012
Liver	0 (0–2)	0 (0–2)	0.752
Cardiovascular	2 (0–4)	0 (0–4)	0.025
Central nervous system	1 (0–4)	0 (0–4)	0.027
Renal	1 (0–4)	1 (0–4)	0.733
Preoperative acute DIC score^a^	3 (1–8)	2 (0–6)	0.482
Serum lactic acid level just before the end of surgery (mmol/L)	5.5 (2.3–15.6)	4.1 (2.2–7.8)	0.132
Operation time (min)	58.5 (23–158)	153 (66–289)	< 0.001
Bleeding (mL)	128 (0–2130)	516 (0–3892)	< 0.001
Fluid and blood transfusion volume during surgery (mL)	3473 (667–7844)	1928 (851–4582)	< 0.001
Use norepinephrine ≥ 0.2 μg/kg/min during surgery, *n* (%)	20 (76.9)	14 (45.2)	0.015
MAP at the start of surgery (mmHg)	68 (33–107)	77 (41–136)	0.143
MAP at the end of source control (mmHg)	65 (51–101)	63 (42–88)	0.804
OAM complications			
No fascia closure, *n* (%)	0 (0)		
Hemorrhagic complications, *n* (%)	0 (0)		
Intestinal fistula, *n* (%)	0 (0)		
Days until closure	2 (1–9)		
28-day mortality, *n* (%)	3 (11.5)	2 (6.5)	0.499
Discharge mortality, *n* (%)	6 (23.1)	5 (16.1)	0.508

Categorical variables are expressed as median (interquartile range)

*DCS* damage control surgery, *SOFA* Sepsis-related Organ Failure Assessment, *DIC* disseminated intravascular coagulation; *MAP* mean artery pressure, *OAM* open abdominal management

^a^Diagnostic criteria for DIC established by the Japanese Association for Acute Medicine

## Discussion

As a result of this study, DCS was performed in patients with more severe septic shock before surgery, but there was no significant difference in mortality of the patients with or without DCS.

All patients who underwent DCS were in septic shock. Although in previous reports, DCS was indicated for cases of sepsis and of septic shock [12, 13], all of our cases were of septic shock, and therefore, we examined only these cases in which DCS was applied. DCS was indicated for acute abdomen at our emergency center for patients with extremely unstable hemodynamics despite appropriate resuscitation before and during surgery. During the study period, although three surgeons in our department determined whether to perform DCS for each patient, we found that the indications for each surgeon had been almost the same.

The SOFA score was significantly higher in the DCS group than in the non-DCS group. In our examination of the details of the preoperative SOFA score, we found significantly higher scores in the DCS group for central nervous system, respiration, cardiovascular, and coagulation, but there was no difference for liver and kidney. Furthermore, DCS was performed for patients with more severe septic shock, and these patients required a significantly large amounts of fluid or blood transfusion or vasoconstrictor (norepinephrine 0.2 μg/kg/min or more) during surgery. Despite the more severe septic shock in the DCS group, there was no significant difference between the mortality rates at 28 days and at discharge of the patients with and without DCS. This result indicated that a DCS strategy could save severely septic patients who would likely suffer a bad outcome without DCS. One of the reasons is operation time. OAM is very simple and rapid, and the initial surgery in a DCS strategy that contains only source control and OAM could significantly reduce the time required for abdominal closure and artificial anus construction. Shortened operation time could also allow intensive care to start earlier. The second reason is the second-look operation. In some cases of non-occlusive mesenteric ischemia, progression of intestinal necrosis occurs after resection [14, 15]. At the planned second-look operation, surgeons have a chance to check necrosis progression, which could be overlooked without OAM. In addition, many patients with septic shock require a large volume of infusion after surgery. Such patients can develop abdominal compartment syndrome (ACS) and subsequent respiratory or circulatory failure if the abdomen is closed in the first surgery [16, 17]. However, abdominal compartment syndrome is not considered to be a concern after surgery when surgery is performed with OAM. Although there are concerns regarding complications of OAM [18, 19], no such complications (bleeding complications, intestinal fistula) occurred at our emergency center. Fascial closure could be accomplished in all patients, and the median time to fascial closure was 2 days.

It was reported that the mortality rate of septic shock ranges from 28 to 41% [20–22], and that of intra-abdominal infection is 36.5% [23], but the mortality rates in our study did not exceed these previously reported rates.

Tobias et al. reported no significantly different mortality in patients undergoing non-traumatic diseases DCS versus non-DCS in their meta-analysis. And they also reported that observed mortality was significantly lower than the expected mortality rate in the DCS [24]. Although we did not calculate expected mortality, our results in this study were similar to their report.

The limitation of this study is that it was a single-center study with a small number of cases.

## Conclusions

In conclusion, this study can help determine the indications for DCS in non-traumatic diseases. DCS can be a very useful technique in the case of severe septic shock. The complications and mortality rates related to OAM were not exacerbated in our study. There are various reports on the indications for DCS in non-trauma patients [25, 26], and our study cannot suggest that DCS should be used for every case of sepsis due to the convenience of OAM. The indication is controversial. And it will be necessary to continue further studies and determine patient conditions for which DCS is useful.

## Data Availability

Not applicable.
